# Supplementary material for: The Effects of Annatto Tocotrienol Supplementation on Cartilage and Subchondral Bone in an Animal Model of Osteoarthritis Induced by Monosodium Iodoacetate
Source: Int J Environ Res Public Health. 2019 Aug 13;16(16):2897. doi: 10.3390/ijerph16162897 (PMC6720523; doi:10.3390/ijerph16162897)
Supplement: Supplementary file 1 [file ijerph-16-02897-s001.pdf]

Supplementary Materials

Table S1. Joint histology scoring of the rats after treatment.

Median (interquartile range)	Pannus Formation	Synovial Hyperplasia	Inflammatory Cells	Cartilage Erosion
Sham	0.00 (0.00)	0.00 (0.00)	0.00 (0.00)	0.00 (0.00)
MIA	2.00 (1.25) [a]	3.00 (1.00) [a]	2.00 (1.25) [a]	3.00 (0.50) [a]
MIA + AnTT 50	1.00 (2.00) [a]	1.50 (1.00) [a]	1.50 (1.00) [a]	1.00 (1.00) [a]
MIA + AnTT 100	0.00 (0.00) [b]	0.00 (0.25) [b,c]	0.00 (0.25) [b,c]	0.50 (1.00) [b]
MIA + AnTT 150	0.00 (0.50)	0.00 (0.50) [b]	0.00 (0.50)	0.00 (1.00) [b]

The letter ‘a’ indicates a significant difference versus the sham group; ‘b’ versus the MIA group; ‘c’ versus the AnTT50 group.

Table S2. A: Serum cartilage and bone remodelling markers of the rats before and after treatment; B: Inter- and intragroup comparison of the serum cartilage and bone remodelling markers of the rats before and after treatment.

**A**								
**Median (SEM)**	**COMP (ng/mL)**		**Hyaluronic Acid (ng/mL)**		**Osteocalcin (ng/mL)**		**CTX-1 (ng/mL)**	
	**Week 0**	**Week 5**	**Week 0**	**Week 5**	**Week 0**	**Week 5**	**Week 0**	**Week 5**
Sham	7.68 ± 0.55	6.90 ± 0.51	690.60 ± 39.85	1329.60 ± 55.89	40.76 ± 1.85	44.58 ± 1.16	21.74 ± 0.46	26.38 ± 1.04
MIA	6.67 ± 0.80	15.48 ± 0.39	629.35 ± 28.62	1796.82 ± 163.34	31.89 ± 0.63	33.44 ± 2.11	18.89 ± 0.39	19.83 ± 0.28
MIA + AnTT 50	4.06 ± 0.74	12.10 ± 1.06	392.12 ± 27.75	1262.12 ± 79.55	37.27 ± 1.57	52.18 ± 3.74	20.18 ± 0.69	24.66 ± 1.32
MIA + AnTT 100	8.49 ± 1.33	7.74 ± 0.33	340.38 ± 47.80	1245.28 ± 47.68	29.99 ± 1.09	31.26 ± 3.69	21.48 ± 0.54	25.01 ± 0.66
MIA + AnTT 150	7.16 ± 1.87	7.50 ± 0.57	356.53 ± 28.97	1265.37 ± 70.02	30.48 ± 1.00	17.07 ± 1.22	21.81 ± 1.12	26.01 ± 0.88
**B**								
**Significance**	**COMP**		**Hyaluronic Acid**		**Osteocalcin**		**CTX-1**	
	**Week 0**	**Week 5**	**Week 0**	**Week 5**	**Week 0**	**Week 5**	**Week 0**	**Week 5**
Sham				*				*
MIA		*a	a	*,a	a	a	a	a
MIA + AnTT 50	a	*,a,b	a,b	*,b	b	*,b		*,b
MIA + AnTT 100	c	b,c	a,b	*,b	a,c	a,c	b	*,b
MIA + AnTT 150		b,c	a,b	*,b	a,c	*,a,b,c,d	b	*,b

The letter ‘a’ indicates significant difference versus the sham group; ‘b’ versus the MIA group; ‘c’ versus the AnTT 50 group; ‘d’ versus the AnTT 100 group; ‘*’ versus week 0.

Table S3. Subchondral bone cellular parameters of the rats after treatment.

Mean (SEM)	Osteoblast Surface	Osteoclast Surface	Eroded Surface	Osteoid Surface	Osteoid Volume
Sham	13.11 ± 0.77	7.22 ± 0.46	3.13 ± 0.29	5.81 ± 0.38	6.68 ± 0.39
MIA	10.52 ± 0.43	9.44 ± 0.73	8.06 ± 1.16 [a]	6.46 ± 0.32	5.76 ± 0.67
MIA + AnTT 50	10.69 ± 1.43	7.30 ± 0.96	6.08 ± 0.69 [a]	6.63 ± 0.80	5.75 ± 0.83
MIA + AnTT 100	11.27 ± 0.21	7.30 ± 035	6.84 ± 0.56 [a]	6.59 ± 1.04	6.89 ± 1.44
MIA + AnTT 150	11.61 ± 0.54	5.46 ± 0.66 [b]	6.43 ± 0.50 [a]	6.80 ± 0.51	4.87 ± 0.74

The letter '[a]' indicates significant difference versus the sham group; '[b]' versus the MIA group.
